# Gut microbiota mediated T cells regulation and autoimmune diseases

**DOI:** 10.3389/fmicb.2024.1477187

**Published:** 2024-12-19

**Authors:** Nabeel Khalid Bhutta, Xiujin Xu, Cuiqin Jian, Yifan Wang, Yi Liu, Jinlyu Sun, Bingnan Han, Shandong Wu, Ansar Javeed

**Affiliations:** ^1^Laboratory of Anti-allergic Functional Molecules, College of Life Sciences and Medicine, Zhejiang Sci-Tech University, Hangzhou, China; ^2^Hangzhou Zheda Dixun Biological Gene Engineering Co., Ltd., Hangzhou, China; ^3^Beijing Key Laboratory of Precision Medicine for Diagnosis and Treatment of Allergic Diseases, Department of Allergy, National Clinical Research Center for Dermatologic and Immunologic Diseases, Peking Union Medical College Hospital, Chinese Academy of Medical Sciences, Peking Union Medical College, Beijing, China

**Keywords:** muciniphila, *Anaerostipes caccae*, Bacteroides sp., Roseburia sp., Blautia sp., *Blautia faecis*, *Clostridium lavalense*, Christensenellaceae sp.

## Abstract

Gut microbiota regulates the immune system, the development and progression of autoimmune diseases (AIDs) and overall health. Recent studies have played a crucial part in understanding the specific role of different gut bacterial strains and their metabolites in different AIDs. Microbial signatures in AIDs are revealed by advanced sequencing and metabolomics studies. Microbes such as *Faecalibacterium prausnitzii, Akkermansia muciniphila, Anaerostipes caccae, Bacteroides* sp.*, Roseburia* sp.*, Blautia* sp.*, Blautia faecis*, *Clostridium lavalense*, *Christensenellaceae* sp.*, Coprococcus* sp.*, Firmicutes* sp.*, Ruminococcaceae* sp.*, Lachnospiraceae* sp.*, Megamonas* sp., *Monoglobus* sp.*, Streptococcus pneumoniae* and *Bifidobacterium* sp. help maintain immune homeostasis; whereas, *Prevotella copri, Ruminococcus gnavus, Lactobacillus salivarius, Enterococcus gallinarum, Elizabeth menigoseptica, Collinsella* sp.*, Escherichia* sp.*, Fusobacterium* sp.*, Enterobacter ludwigii, Enterobacteriaceae* sp.*, Proteobacteria*, *Porphyromonas gingivalis*, *Porphyromonas nigrescens*, *Dorea* sp., and *Clostridium* sp. cause immuno-pathogenesis. A complex web of interactions is revealed by understanding the influence of gut microbiota on immune cells and various T cell subsets such as CD4+ T cells, CD8+ T cells, natural killer T cells, γδ T cells, etc. Certain AIDs, including rheumatoid arthritis, diabetes mellitus, atopic asthma, inflammatory bowel disease and non-alcoholic fatty liver disease exhibit a state of dysbiosis, characterized by alterations in microbial diversity and relative abundance of specific taxa. This review summarizes recent developments in understanding the role of certain microbiota composition in specific AIDs, and the factors affecting specific regulatory T cells through certain microbial metabolites and also focuses the potential application and therapeutic significance of gut microbiota-based interventions as novel adjunctive therapies for AIDs. Further research to determine the precise association of each gut bacterial strain in specific diseases is required.

## Graphical abstract

The importance of the gut microbiota in immunological control and health is described in this graphical abstract, which contrasts its role in immune tolerance during homeostatic condition with that of autoimmune inflammation. It emphasizes the role of microbiota in T lymphocyte subpopulations, Gut-associated lymphoid tissue (GALT) development, and the synthesis of SCFAs and antimicrobial peptides. It also outlines how important dendritic cells (DCs) are in facilitating the synthesis of commensal-specific IgA via plasma cells, in order to maintain gut homeostasis. While, dysbiotic microbiota creates antigens in inflammatory conditions that lead to adverse immune responses, impairing mucosal integrity and exacerbating autoimmune diseases.
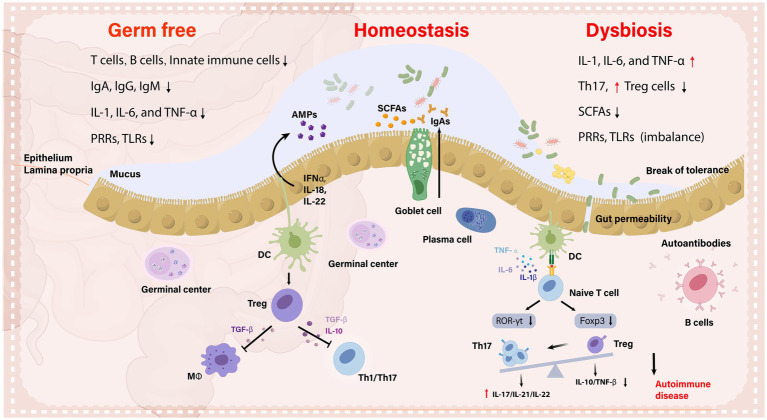

## Introduction

**Figure 1:**
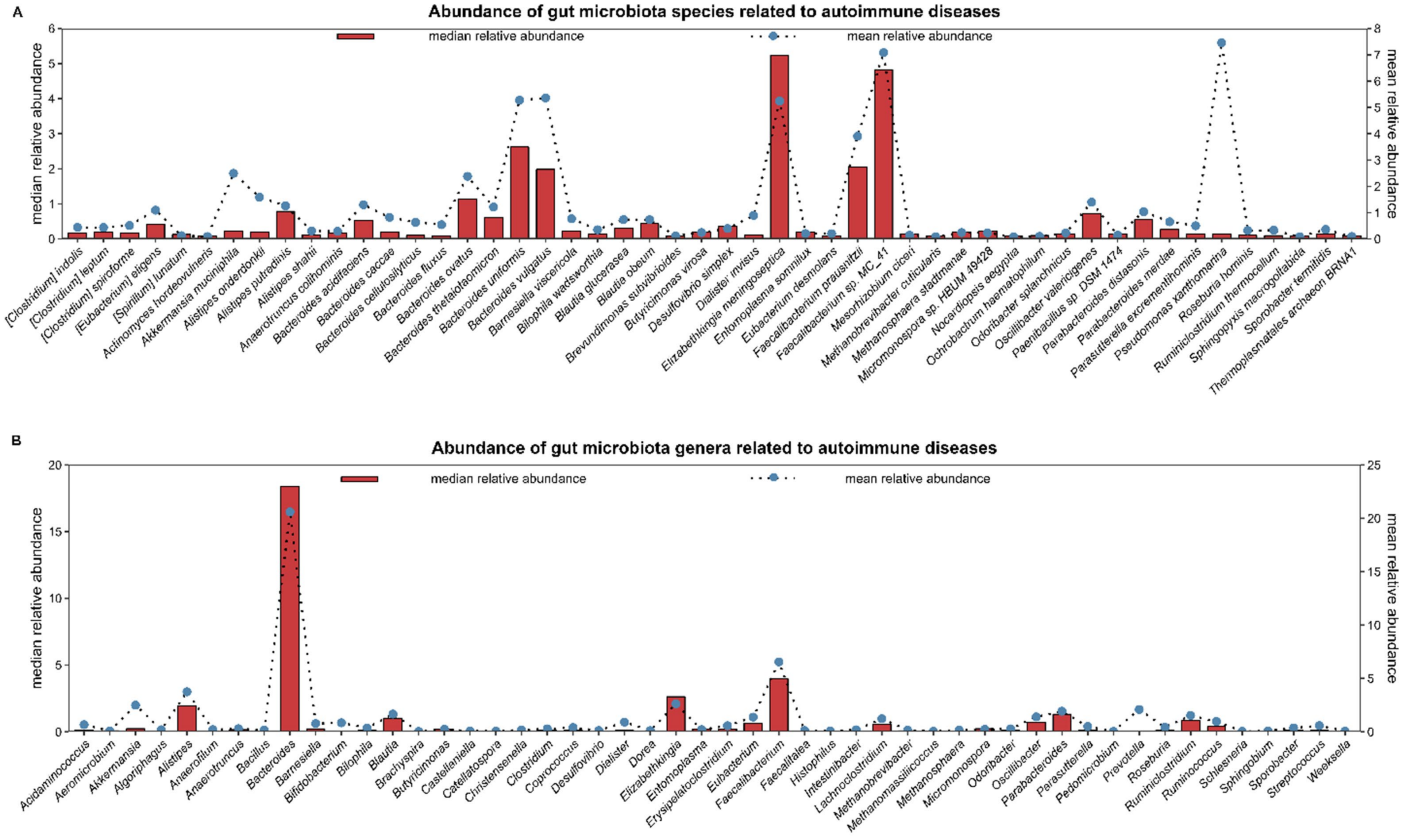
The American Gut project is the largest crowdsourced citizen science project to date. Fecal, oral, skin, and other body site samples collected from thousands of participants represent the largest human microbiome cohort in existence. It is the PRJEB11419 project on NCBI (National Center for Biotechnology Information). In the PRJEB11419 project, a total of 1,053 samples were related to the phenotype of autoimmune diseases. In the GMrepo database, 553 samples were selected from the 1,053 samples for research, and 3,095 species and 1,016 genera were found. In figure, the top 50 species/genera of gut microbiota are selected in descending order based on the median relative abundance. **(A)** shows 50 species; **(B)** shows 50 genera. Abundance: mean/median relative abundance of a species/genus in all samples of autoimmune diseases.

It is well recognized that certain environmental factors cause autoimmune diseases (AIDs) in individuals having genetic predisposition, and there is evidence linking epigenetic dysregulation to AIDs pathogenesis (Araki and Mimura, 2018; Zhao et al., 2019). Numerous environmental factors, including pesticides, heavy metals and smoking have been linked to AIDs (Khan and Wang, 2018). In recent times, the gut microbiome is reported to be linked with AIDs due to its ability to cause immune dysregulatory and pro-inflammatory effects through microbiome dysbiosis (Dehner et al., 2019). The American gut project is recognized as largest crowdsourced citizen science project till now. The largest known human microbiome cohort was created from samples taken from thousands of participants via oral, skin, feces and other body sites. It is the PRJEB11419 project on NCBI (National Center for Biotechnology Information). In this project, a total of 1053 samples were related to the phenotype of AIDs. In the GMrepo database, 553 samples were selected from the 1053 samples for research, and 3095 species and 1016 genera were found (Wu et al., 2020). We sorted top 50 species/genera in descending order of median relative abundance and found that the species with higher median relative abundance include *Elizabeth menigoseptica*, *Faecalibacterium sp.* MC_41, etc., while the genera with higher median relative abundance include *Bacteroides*, *Faecalibacterium*, etc. (Figure 1). This large-scale, crowdsourced microbiome analysis offers insights about dysbiosis’ relation with the development of AIDs and may open avenues for personalized microbiome-based diagnostics and treatments.

The immune homeostasis disruption leads to an increase in effector Th1, Th17, and plasma cell populations. Antigen-presenting cells (APCs), predominantly the DCs and macrophages, are significant because they sample antigens and initiate an inflammatory response. These APCs are able to transport toxins and antigens derived by the luminal microbiota to effector B and T cells, activating them in the process as part of the interaction of immune system with gut microbiota (Farache et al., 2013). Furthermore, antimicrobial peptides (AMPs), are essential for separating the gut bacteria from epithelium (Bevins and Salzman, 2011). AMPs are innate immuno-effectors that are released by epithelial cells in response to cytokines (IFNα, IL-18, and IL-22) produced by macrophages and DCs against microbial antigens (Mergaert, 2018). In a healthy state, when the immune system and gut microbiota work together, they encourage the immuno-modulatory T-reg cell differentiation and proliferation while suppressing the pro-inflammatory pathways (Mowat, 2018). However, abnormalities in the gut microbiota composition can cause harmful autoimmune reactions, especially in individuals having genetically predisposition. An illustration of this is the activation of Th17, which is linked to specific gut microbiota species such as *Prevotella copri, Bifidobacterium adolescentis* and *Enterococcus gallinarum* (Manfredo Vieira et al., 2018; Asquith et al., 2016; Tan et al., 2016).

**Figure 2:**
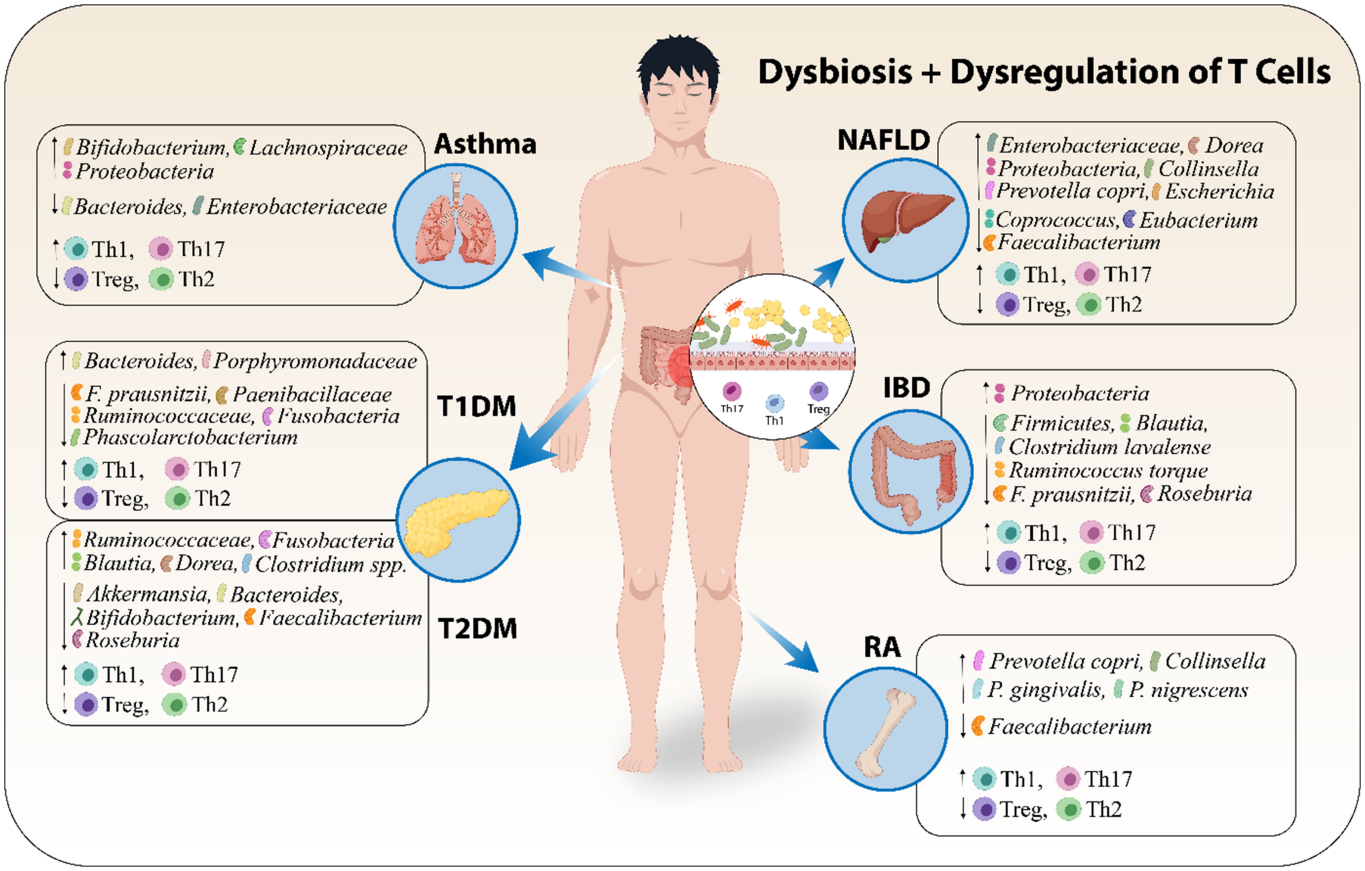
Dysbiosis along with dysregulation of T cells leads to certain AIDs. Abbreviations include Type 1 Diabetes Mellitus (T1DM), Type 2 Diabetes Mellitus (T2DM), and Non-Alcoholic Fatty Liver Disease (NAFLD), Inflammatory Bowel Disease (IBD), Rheumatoid Arthritis (RA).

Metabolites derived from the gut microbiota, like SCFAs, possess the ability to alter a cell's metabolic state, hence inducing regulatory B cells and preventing pentanoate-induced Th17 cell production (Luu et al., 2019). Furthermore, breakdown products from tryptophan can cause an increase in intraepithelial CD4+ CD8+ T cells, microbiota-generated ATP can stimulate Th17 cell proliferation, and polysaccharides derived from bacteria can stimulate regulatory T cells (Ahern and Maloy, 2020; Wiertsema et al., 2021). Moreover, the over-activation of plasma cells that produce antibodies is one way by which gut microbiota influence the immune system and contribute toward autoimmunity (Ma et al., 2019). Figure 2 shows that dysbiosis along with dysregulation of T cells leads to certain AIDs.

A change in our understanding of the etiology of different inflammatory disorders has resulted from the discovery of the gut microbiota involvement in the remodeling of immune cells' epigenome and it has opened new avenues for therapeutic mechanisms. The immune system's reaction to AIDs is complicated, and both B and T cells are essential to the pathophysiology of these conditions. While, gut microbiota has long been known to affect B cell functions and to regulate immune responses, there is mounting evidence that T cells, specifically T helper cells (Th1, Th2 and Th17) and regulatory T cells (Tregs) are key contributors to the immune dysregulation seen in AIDs. T cell subsets differentiation, including helper T cells (Th1, Th2 and Th17) and Treg cells, is associated with particular microbiota species (Mazmanian et al., 2005; Gaboriau-Routhiau et al., 2009; Atarashi et al., 2011). Furthermore, the gut microbiota's metabolic byproducts, e.g. short-chain fatty acids (SCFAs), have influence on the T cells activation and differentiation (Saade et al., 2022). Numerous factors, including metabolic activities, immune dysregulation and pro-inflammatory pathways can cause the gut microbiota dysbiosis. T cells-related disorders such as RA, type 1 and type 2 diabetes mellitus, atopic asthma, IBD and NAFLD are all influenced by dysbiosis (Valdes et al., 2018; Ananthakrishnan et al., 2018; Cenit et al., 2017). Hence, in this article, we have reviewed the gut microbiota relation with AIDs, how gut microbiota manipulates different T cells functions and contributes toward immuno-pathogenesis of different AIDs and also discussed the gut microbiota-based interventions as innovative therapeutic options for AIDs.

## Material and methodology for literature survey

This review contains original research articles published in English language from 2004 to 2024, of which mostly were published after 2015. During the year of 2024, different database searches are performed on PubMed, Web of Science, Elsevier, MDPI and Springer Link etc., using keywords such as “gut microbiota” + “autoimmune diseases”, “inflammatory bowel disease”, “Treg cells function”, “CD4+ T cells”, “CD8+ T cells”, “γδ T cells”, “natural killer T cells”, “Type 1 diabetes mellitus”, “Type 2 diabetes mellitus”, “rheumatoid arthritis”, “atopic asthma”, and some other combined keyword searches. We only included studies that were published in well renowned journals of relevant topics and are completely consistent with the subject of this review. The purpose of this review article is to summarize the recent researches on autoimmune diseases and their relation with gut microbiota to provide enough information relevant to the topic of this review article. In order to facilitate readers, we have added mechanisms based figures for graphical presentation of concept and clear understanding.

## Gut microbiota’s association with AIDs

The genetic susceptibilities of the individual cannot entirely describe the pathophysiology of human diseases, which emphasizes the need to investigate the roles played by environmental factors. Increasing research has demonstrated the link between aberrant changes in gut microbiota and a variety of disease types, including autoimmune disorders [such as spondyloarthritis (SpA), systemic lupus erythematosus (SLE), Sjögren’s syndrome (pSS), Multiple Sclerosis (MS), Inflammatory bowel disease (IBD), Rheumatoid arthritis (RA), etc] which is corroborated by our understanding of the pathogenic activities of gut microbiota (Yin et al., 2020; Thomas et al., 2019). The microbiota, which is found on the skin and mucosa of the host, coevolves with the host and is most prevalent in the gastrointestinal tract. These microbes contain roughly 150 times greater metagenome, or genomic contents in comparison to humans (Qin et al., 2010). The gut microbiota and immune system work together in a suitable manner to combine innate and adaptive immunity in such a way that selects, modifies, and terminates responses in the most appropriate manner. Immune system and gut bacterial communities correlate with each other in maintaining the healthy environment. Disturbance in either of these results in certain autoimmune disorders.

With time, the advancements in high-throughput DNA sequencing have made investigation of the complex gut microbes signature much easier, especially those microbes which are difficult to culture in vitro. Since metagenomic research was initially used to study the gut microbiota in gastrointestinal disorders such as colorectal cancer and IBD, now been extended to study the gut microbiota in AIDs such as SpA (Yin et al., 2020; Wen et al., 2017; Zhou et al., 2020), SLE (Li et al., 2019; Azzouz et al., 2019; Chen et al., 2019), MS (Jangi et al., 2016), RA (Chiang et al., 2019; Alpizar-Rodriguez et al., 2019; Zhang et al., 2015; Scher et al., 2013) and pSS (van der Meulen et al., 2019; Mandl et al., 2017). These techniques have uncovered dysbiosis patterns and linked it to immune regulation. However, limitations like insufficient strain-level resolution and the need for functional validation highlight the importance of integrating multi-omics approaches to better understand gut microbiota’s role in AIDs. The gut microbiota of the individuals having AIDs differs significantly in comparison to healthy people. For example, species such as *Lactobacillus salivarius, Ruminococcus gnavus* and *Prevotella copri* have been linked to the pathogenesis of AIDs. However, The complex relationships between the host and microbes make it difficult to translate data from animal models showing the immune-regulatory behavior of particular strains of bacteria and their metabolites into clinical implications (Hui et al., 2019; Evans-Marin et al., 2018; Zegarra-Ruiz et al., 2019; Manfredo Vieira et al., 2018). New biomarkers and treatment options can be obtained by examining temporal dynamics, transgenerational impacts, and microbial metabolite-induced epigenetic changes. Validating this hypothesis can provide information about tailored microbiota-based therapies for managing and preventing AIDs.

## Gut microbiota disturbance and development of AIDs pathogenesis

One of the greatest interfaces (250–400 m^2^) in the human body exists between the host, antigens and environmental conditions in the gastrointestinal (GI) tract (Thursby and Juge, 2017). The human GI is home to greatest microbial community known as the "gut microbiota," that is made up of a wide variety of microorganisms, such as archaea, bacteria and eukarya, that have coevolved over millions of years with their host to form a complex and mutually beneficial symbiotic relationship (Neish, 2009). Various immune cells, including innate lymphocytes, macrophages, and DCs, make up the gut-associated lymphoid tissue (GALT), which is the first line of defense against pathogens (Bellocchi et al., 2019). The gut microbiota has been extensively studied in relation to human disease, and knowledge about its composition and functions has grown rapidly (Piccioni et al., 2022). The gut microbiota is mainly responsible for preserving the equilibrium between host defense and immune tolerance, and it is also a major factor in the development of the immune system (Zhang et al., 2023; Jiao et al., 2020; Piccioni et al., 2022). Interestingly, inadequate exposure to microbes during early life has been associated with an increased risk of AIDs. This highlights how important the early gut microbiota development is in influencing the immune system of their host (Stiemsma et al., 2015). The relationship between host immunity and gut microbiota suggests that AIDs are partly caused by dysbiosis of the gut microbiota.

The gut microbiota composition of individuals having AIDs differed significantly from that of healthy ones as demonstrated by advanced techniques like whole-genome sequencing and metatranscriptomic analysis, suggesting an imbalance in the gut ecosystem. The gut microbiota was found to be less diverse in cases of celiac disease, irritable bowel syndrome (Jackson et al., 2018) and several AIDs including RA, SpA, pSS, and SLE (Chiang et al., 2019; Azzouz et al., 2019; Wen et al., 2017; van der Meulen et al., 2019). It was indicated by a decrease in taxonomical richness and evenness in the microbiota. Therefore, there is need to explore whether specific microbial signatures, such as reduced levels of anti-inflammatory species or overabundance of pro-inflammatory taxa, correlate with disease activity and prognosis in different AIDs.

The presence of particular microbial taxa may serve as a biomarker for evaluating the severity of AIDs. For example, elevated *R. gnavus* was linked to lupus nephritis (Azzouz et al., 2019), while elevated *L. salivarius* was linked to higher disease activity in patients with SLE or RA (Zhang et al., 2015; Chen et al., 2019; Liu et al., 2013). Several studies highlight, *R. gnavus*, a bacteria known to degrade mucin, was in higher abundance in the feces of SLE, SpA, and RA patients. RA and ankylosing spondylitis (AS) patients had higher abundances of *P. copri* in their feces (Alpizar-Rodriguez et al., 2019; Scher et al., 2013; Wen et al., 2017; Zhou et al., 2020). On the other hand, a few bacteria, like *Faecalibacterium prausnitzii*, were found in lower amounts in AS and pSS patients (Yin et al., 2020; Stewart et al., 2018), indicating possible beneficial functions. In a recent study, Wang et al. reported, a significant decrease in the Firmicutes/Bacteroidetes (F/B) ratio in Lupus nephritis patients (Wang et al., 2023a). In a study reported, the *Firmicutes* in the microbial diversity was decreased in RA patients who had anti-citrullinated protein antibody (ACPA), while certain strains such as *Blautia, Clostridiales* and *Akkermansia* showed significant enrichment (Chiang et al., 2019).

Furthermore, Elevated number of pro-inflammatory bacteria like *Akkermansia spp.* and reduced numbers of *Lachnospiraceae* and *Faecalibacterium* (which produce anti-inflammatory chemicals) have been linked to the pathophysiology of MS (Jangi et al., 2016) *A. muciniphila* and *Acinetobacter calcoaceticus*, which were isolated from individuals suffering from MS and incubated in monocolonized mice, induced pro-inflammatory responses and *Parabacteroides distasonis* stimulated anti-inflammatory human IL-10+FoxP3+ cells and CD4+CD25+ T cells (Cekanaviciute et al., 2017; Sadeghpour Heravi, 2024). However, the predictive power of microbial species for disease severity is still unknown. Standardization of study methodologies and further research on the regulatory roles of different microbes across various AIDs is needed to address these inconsistencies.

Whereas the host immune system regulates microbial ecology, the microbiota also generates a wide range of biochemical active metabolites, including SCFAs and tryptophan metabolites, which impact the immune system's development and function. Studies on lupus-prone mice have supported the functional analysis found in patients with systemic lupus erythematosus (SLE), which indicated a rise in the biosynthesis of tryptophan, ornithine and arginine with a reduction in the biosynthesis of branched-chain amino acids (Chen et al., 2019). Abnormal pathways in patients with ankylosing spondylitis include degradation of glycosaminoglycan, lipopolysaccharide biosynthesis, and oxidative phosphorylation (Zhou et al., 2020).

**Figure 3:**
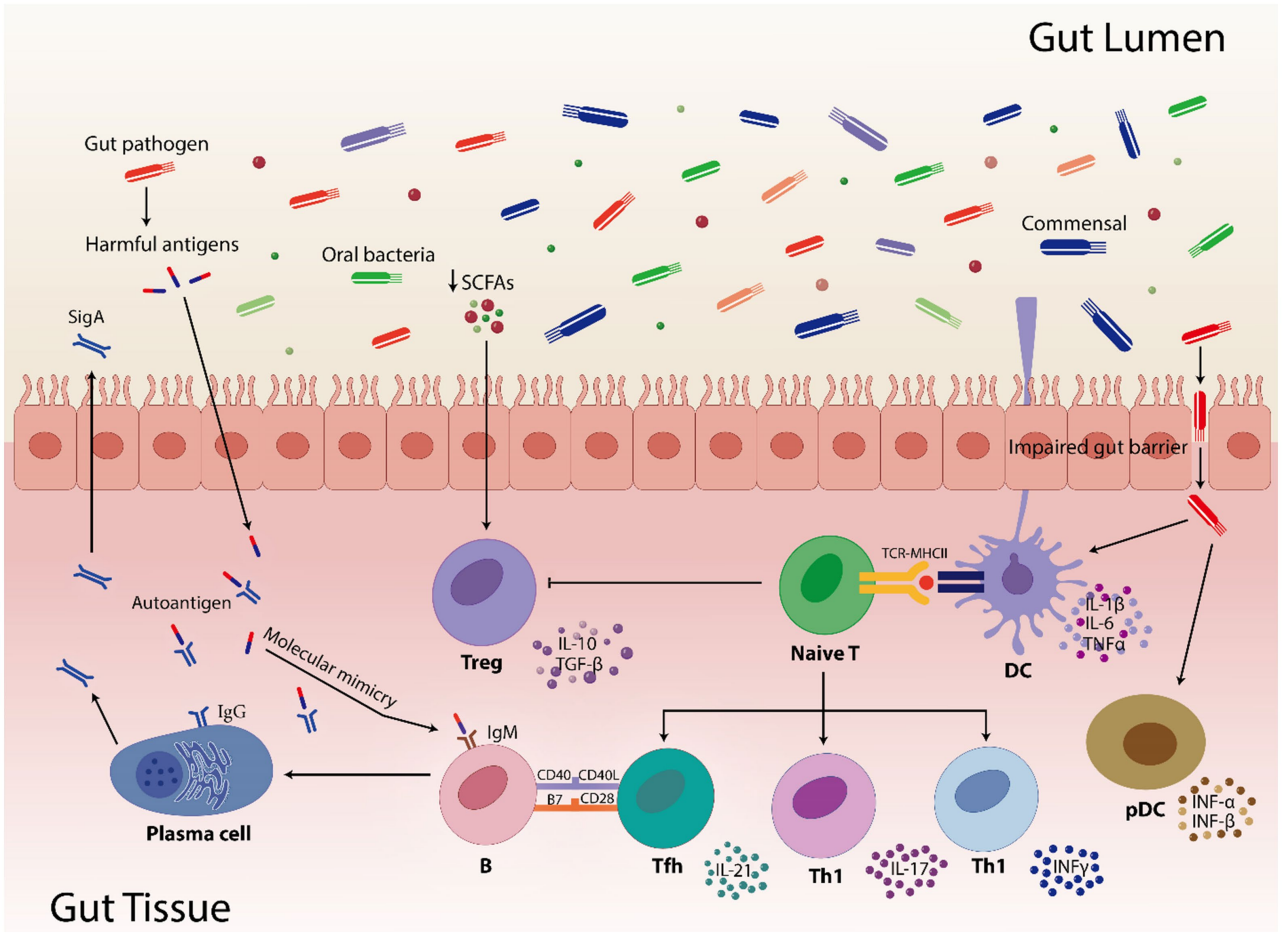
Highlights how gut pathogens and commensal bacteria can influence the gut immunity. Short-chain fatty acids (SCFAs) are involved in controlling immunological responses, including the activation of regulatory T cells (Treg). In pathogenic condition, SCFAs numbers are reduced which results in reduced activation of Treg cells. Furthermore, compromised gut barrier function facilitates the translocation of commensals and pathogens by inducing pro-inflammatory cytokines (IL-1β, IL-6, TNF*α*, INF-α, and INF-β) via dendritic cells (DC) and plasmacytoid dendritic cells (pDC). The TCR-MHCII contact on DCs activates naive T cells, which thereafter differentiate into diverse T helper cell subsets (Tfh, Th1, and Th17) and contribute to the gut’s immunological environment. Moreover, molecular mimicry is caused when microbial antigens structurally resemble the human autoantigens leading to B cell activation that use the assistance of Tfh cells to produces both protective sIgA and, in dysregulated states, pathogenic autoantibodies. In addition, impaired gut barrier facilitates the transfer of microbial antigens, which can result in immunological cross-reactions where T and B cells may mistakenly target host tissues by activating the T cell subsets and pro-inflammatory cytokines. These interactions highlight how the gut microbiome shapes autoimmune susceptibilities, especially in hosts who are genetically susceptible.

Moreover, when several immune pathways were abnormally triggered due to dysbiosis, anti-inflammatory cytokines (like IL-4, IL-1ra, TGF-β, IL-10, IL-13, etc.) decreased and pro-inflammatory cytokines (like IL-6, IL-12, TNF-α, IFN-γ, IL-1β, IL-17, etc.) increased (Zhang et al., 2014). Therefore, it is crucial to conduct longitudinal research and functionally characterize gut microbial species. Translational research has to find a way to connect human and animal studies, find biomarkers based on microbiome, and develop focused treatments. Furthermore, investigating how lifestyle of an individual and environmental variables affect gut microbiota will help in developing preventative measures and improvement of the disease treatment. This will also clarify the complex interactions among host genetics, immunotypes and gut microbiota. Figure 3 describes the mechanism by which disturbed gut microbiota leads to AIDs and Table 1 highlights the increase or decrease in various bacterial species involved in different autoimmune diseases and their role toward diseases regulation.

**Table 1:** Variations in population of gut microbes correlated with different autoimmune diseases and their involvement.

Autoimmune Diseases	Increased and decreased gut microbiota species	Involvement of microbial species in certain diseases
Rheumatoid Arthritis (RA)	↑ *Prevotella copri* (Alpizar-Rodriguez et al., 2019; Scher et al., 2013; Wen et al., 2017; Zhou et al., 2020), *Ruminococcus gnavus, Lactobacillus salivarius* (Zhang et al., 2015; Chen et al., 2019; Liu et al., 2013), *Akkermansia muciniphila* (Chiang et al., 2019) ↓ *Faecalibacterium prausnitzii*	*Prevotella copri* and *Ruminococcus gnavus* are involved in increased inflammation and disease activity. *Lactobacillus salivarius* is associated to higher disease activity. *Faecalibacterium prausnitzii* is anti-inflammatory.
Systemic Lupus Erythematosus (SLE)	↑ *Ruminococcus gnavus, Lactobacillus salivarius* (Zhang et al., 2015, Chen et al., 2019, Liu et al., 2013) ↓ *Faecalibacterium prausnitzii*	*Ruminococcus gnavus* and *Lactobacillus salivarius* are involved in disease enhancement. Activity. *Akkermansia muciniphila* role is variable.
Spondyloarthritis (SpA)	↑ *Prevotella copri, Ruminococcus gnavus* ↓ *Faecalibacterium prausnitzii*	*Prevotella copri* is involved in disease. *Ruminococcus gnavus* is involved in inflammation. *Faecalibacterium prausnitzii* is beneficial.
Primary Sjögren’s Syndrome (pSS) Multiple Sclerosis (MS)	↑ *Akkermansia muciniphila* ↓ *Faecalibacterium prausnitzii* ↑ *Akkermansia muciniphila, Acinetobacter calcoaceticus* (Cekanaviciute et al., 2017) *↓ Lachnospiraceae, Faecalibacterium* (Jangi et al., 2016), *Parabacteroides distasonis* (Cekanaviciute et al., 2017)	*Akkermansia muciniphila* role is complex and may vary. *Faecalibacterium prausnitzii* generally plays anti-inflammatory role. *Akkermansia muciniphila* and *Acinetobacter calcoaceticus* are pro-inflammatory. *Lachnospiraceae*, *Faecalibacterium* and *Parabacteroides distasonis* are anti-inflammatory.
Ankylosing Spondylitis (AS)	↑ *Prevotella copri* (Alpizar-Rodriguez et al., 2019, Scher et al., 2013, Wen et al., 2017, Zhou et al., 2020)*, Akkermansia muciniphila* ↓ *Faecalibacterium prausnitzii*	*Prevotella copri* is linked to disease. *Akkermansia muciniphila* role may vary. *Faecalibacterium prausnitzii* is beneficial.
Lupus Nephritis (LN)	↑ *Ruminococcus gnavus* (Azzouz et al., 2019), *Bacteroidetes* (Wang et al., 2023a) *↓ Firmicutes* (Wang et al., 2023a)	*Ruminococcus gnavus* is associated with severe kidney involvement in Lupus Nephritis, while *bacteoidetes* disrupt gut homeostasis and contribute to immune system dysregulation that leads to lupus nephritis. *Firmicutes* plays anti-inflammatory role.

**↑: increased; ↓: decreased levels of certain microbial populations.**

## Gut microbiota-mediated manipulation of T cells function

Complex interactions between intestinal mucosal immunity and gut microbiota have a wide range of impacts on disease processes and homeostasis (Zhang and Pan, 2020). The human immune system and the microbiota mainly interact in GI tract. The innate immunity serves as first line of defense while the adaptive immunity precisely targets pathogens and creates immunological memory (Thakur et al., 2019). The gut microbiota has a major impact on disease states and immunological homeostasis by influencing the differentiation and function of different T cell subsets. Inflammation and immunological tolerance can be impacted by the promotion or inhibition of T helper (Th1, Th2, Th17), regulatory T (Treg), cytotoxic CD8+ T, natural killer T (NKT) and γδ T cells responses by particular bacterial species and their metabolites. These interactions are frequently upset by dysbiosis, which results in immunological abnormalities that fuel autoimmune disorders (AIDs).

### CD4+ T cells

**Figure 4:**
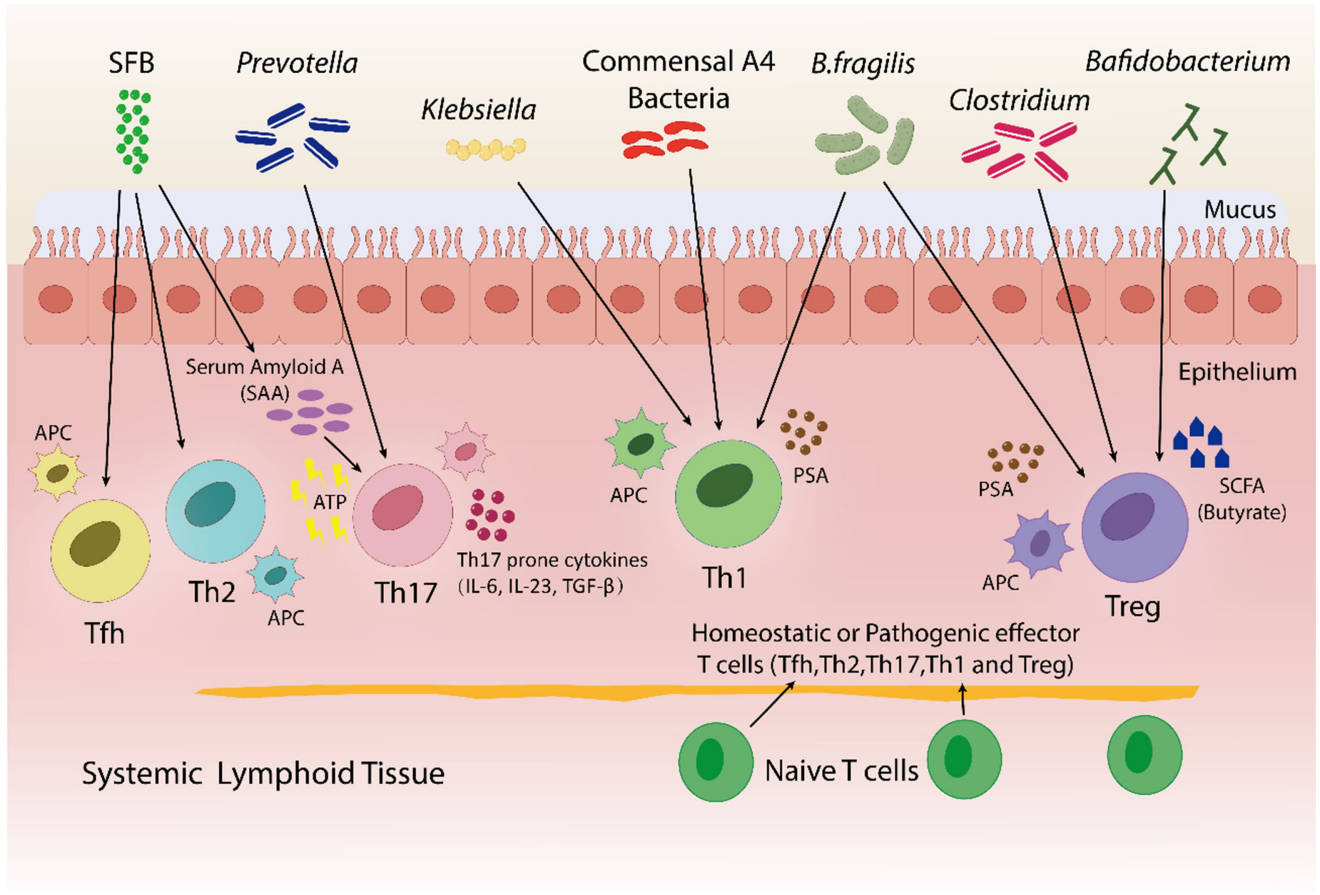
In both pathogenic and homeostatic settings, the microbiota is an essential factor in guiding T-cell differentiation. By using a bacterial product called PSA in a PSA-dependent pathway, *B. fragilis* promotes the growth of Th1-associated immune responses in germfree mice. *Klebsiella* and commensal A4 bacteria also induces Th1 cells. The proliferation of regulatory T (Treg) cells is encouraged by *B. fragilis*. By utilizing innate cells to produce either serum amyloid A (SAA) or adenosine 5′-triphosphate (ATP), SFB trigger a Th17 immune response. SFB also promotes the Tfh and Th2 cell production. *Prevotella* induces Th17 cells. *Bafidobacterium* produces SCFA, which stimulates the generation of Treg cells. *Clostridium* aids in the development of immunotolerance by promoting the production of Treg cells. Furthermore, within a particular tissue environment, systemic T cells can give rise to tissue-specific T cells through the activation of APCs by bacterial antigens.

Adaptive immune responses mainly depend on CD4+ T helper cell differentiation into subsets (such as Tfhs, Th1s, Th2s, Th17s, Tregs, and other subtypes) with a variety of effector activities especially for the defense of host against pathogens (Saravia et al., 2019; Li et al., 2022b). Various studies highlight the influence of the gut microbiota on CD4+ T cells differentiation. Some species of *Klebsiella* genera, including *K. pneumoniae* and *K. aeromobilis*, cause Th1 cell responses in the gastrointestinal tract. *Klebsiella* colonization increases Th1 cell proliferation in the intestines of germ-free mice, thereby increasing the number of these cells (Atarashi et al., 2017) (Figure 4). Probiotic bacteria, especially *Lactobacillus* strains like *L. salivarius* (Ren et al., 2019) and *L. plantarum* (Matsusaki et al., 2016; Takeda et al., 2011), increase the Th1 cytokines [interferon-gamma (IFNγ) and tumor necrosis factor-alpha (TNFα)] production, while, inflammatory immune responses by Th1 cells were inhibited and antigen-specific oral tolerance was enhanced in arthritic mice treated with *L. casei* (So et al., 2008).

A research indicated that germfree mice exhibits a preference for Th2 cell responses; however, the introduction of polysaccharide A (PSA) derived from *Bacteroides fragilis* corrects this imbalance by favoring Th1 cell responses (Mazmanian et al., 2005). PSA via the toll-like receptor (TLR2) interacts with DCs to stimulate the release of IL-12. IL-12 causes STAT 4 activation, which results in differentiation of CD4+ T cell into Th1 cells that produce IFN-γ (Wang et al., 2006). In healthy conditions, the differentiation of Th1 cells specific to *Klebsiella* can be controlled without causing severe inflammation in the gut. In contrast, during dysbiosis, *Klebsiella* dominance may induce severe gut inflammation through the induction of Th1 cell differentiation, as evidenced by more prevalence of *Klebsiella* species in fecal samples taken from IBD patients in comparison to healthy individuals (Lloyd-Price et al., 2019). Thus, the Th1/Th2 response can be balanced by the introduction of engineered *B. fragilis* strains that could overproduce polysaccharide A (PSA) and therefore, prevent *Klebsiella*-induced Th1 cell differentiation and severe gut inflammation in IBD patients.

Th2 cells generate antibodies to combat infections, proliferate and develop into plasma cells, and also stimulate B cells (Chen et al., 2021a). Certain types of bacteria, like A4 bacteria in the *Lachnospiraceae* family, prevent Th2 cells from differentiating and being active by making DCs based TGF-β (Wu et al., 2016). It has been determined that *B. fragilis* and *lactobacillus* strains inhibit Th2 activity, thereby enhancing Th1 activity (Mazmanian et al., 2005; Takeda et al., 2011; Won et al., 2011). Bamias et al. reported that the Th2 response was induced by commensal bacteria in the chronic phase of Crohn's disease (CD)-like ileitis in SAMP1/YitFc mouse models, and symptoms were also deteriorated (Bamias et al., 2007). A symbiotic combination of A4 bacteria, *B. fragilis* and *Lactobacillus* strains can be administered to Crohn’s disease patients, that may inhibit Th2 cell differentiation and activity, hence lowering chronic inflammation and enhancing symptom management.

Th17 cells stimulate neutrophils, produce and secrete a variety of inflammatory cytokines, including IL-17 and IL-22, and infiltrate lesions to increase the inflammation. Retinoic acid-related orphan receptor (RORγt), an important transcription factor, facilitates Th17 cell development and Th17 cytokine production (Nalbant and Eskier, 2016; Chen and Tang, 2021). Interestingly, Th17 cells are not present in germfree mice, rather, they are induced when bacteria colonize the mice (Lee and Kim, 2017). Certain bacteria, like gram-positive bacteria and SFB, have been found to be inducers of Th17 cell proliferation. Serum amyloid A (SAA), elevates when SFB typically penetrates the mucus layer and adheres to epithelial cells. SAA markedly enhances the Naive CD4+ T cell differentiation into Th17 cells (Atarashi et al., 2015; Li et al., 2022b).

Furthermore, the function of SFB such as *Candidatus Arthromitus*, and *Prevotella* has been reported in fostering strong Th17 cell proliferation along with the release of IL-17 and IL-22 (Atarashi et al., 2015; Farkas et al., 2015; Ivanov et al., 2009; Schnupf et al., 2015). In a study, *P. gingivalis* was introduced into a collagen-induced arthritis (CIA) mice model, found elevation in joint disease severity which was linked to systemic proinflammatory cytokine profiles that indicated Th17 pathway activation (Moen et al., 2006). A study reported a secondary bile acid produced by microbe to have a negative effect on Th17 cell differentiation. Th17 transcription factor RORγt (retinoic acid-related orphan receptor gamma t) was blocked by 3-oxolithocholic acid along with one of thirty diverse primary and secondary types of bile acids and the decrease in the differentiation of Th17 cells was observed in SPF mice (Hang et al., 2019). The exact mechanism by which certain bacteria induce intestinal Th17 differentiation is still unknown, thus, research must go into modifying gut microbiota to regulate the differentiation of Th17 cells and create innovative remedies for AIDs. Another promising therapeutic approach is to look into the interactions of bile acids with Th17 cells differentiation.

Treg cells are essential for the prevention of AIDs because they preserve immune homeostasis, control immune responses and promote tolerance to harmless antigens (Dominguez-Villar and Hafler, 2018). *B. fragilis* is able to trigger to generate large populations of Treg cells by producing PSA, which in turn causes Foxp3+ Treg cells to develop and produce IL-10 (Round and Mazmanian, 2010) as shown in Figure 4. Furthermore, adenosine and inosine, two bacterial metabolites, can bind with the adenosine A2A receptor (A2AR) on T cells, increasing the activity of Treg cell and suppressing the inflammatory responses of Th1 and Th17 (Liu et al., 2018; Wang et al., 2024). In a recent study Wang et al. found, *Megamonas*, *Monoglobus*, and *Prevotella* relative abundances were favorably connected with cytokine levels and CD4+ T cell counts; on the other hand, the T helper (Th17)/Treg ratio and the relative abundance of regulatory T cells (Tregs) were correlated negatively with RA disease activity (Wang et al., 2022b). Sun et al. reported the change in the composition of gut microbiota's by *Bifidobacterium* colonization. This improved the suppressive Treg cells function by encouraging their mitochondrial activity (Sun et al., 2020). Certain *Lactobacillus* strains, like *Lacticaseibacillus casei*, affect the differentiation and activity of T cells, resulting in the development of Treg cells and the release of IL-10 (Smits et al., 2005). *L. acidophilus* strain L-92 demonstrated related outcomes in BALB/c mice. L-92, when administered orally, led to a rise in the expression of Foxp3, TGF-β and IL-10 in mice under conditions of allergic contact dermatitis (ACD). This established the concept that L-92 mitigates ACD by increasing the proliferation of Treg cells, and Th1 and Th2 responses (Shah et al., 2012). A study conducted on in BALB/c mice, demonstrated that *L. reuteri* reduces allergic airway reactions (Forsythe et al., 2007) thereby, increases Foxp3 expression (Karimi et al., 2009), which positively affects the proliferation of Treg cell and reduces the intensity of AIDs. It has been shown that a different *Lactobacillus* strain, *L. murinus* regulates the small intestine's RORγt+ Treg cells, which reduces pulmonary inflammation caused by *Mycobacterium tuberculosis* infection (Bernard-Raichon et al., 2021).

Zhang et al. demonstrated that antibiotic-induced dysbiosis such as ampicillin, decreases Treg cell production and interferes with Th1 responses to infection caused by bacteria (Zhang and Bevan, 2011). Treg cell production and activities can also be affected by modifications in the microbiota, which can be caused by a variety of factors as Figure 4 illustrates, *clostridium* species are thought to have an effect on Treg cells, whose dysregulation can result in autoimmunity. These studies highlight, certain bacteria, such as *B. fragilis* and specific strains of *Bifidobacterium* and *Lactobacillus*, increase the production and activity of Treg cells, in result, reduce inflammatory responses and disease severity. Furthermore, antibiotics-induced dysbiosis can disrupt the production of Treg cells, that highlights the role healthy microbiota play in immune regulation.

### CD8+ T cells

CD8+T cells also referred as cytotoxic T lymphocytes (CTLs), are essential components of the immune system because they specifically target and destroy malignant cells. Shimokawa et al. reported, Nematode-derived trehalose alters the gut microbiota and elevates the number of *Ruminococcus* species, which stimulates CD8+ T cells (Shimokawa et al., 2020). To enhance the effectiveness of immune-based therapies, there is need to understand how gut microbiota modulation, induced by dietary components like nematode-derived trehalose or probiotics, influences systemic immune responses like CD8+ T cell activation and infiltration of various tissues. A research discovered an association of commensal strains, predominantly *Bacteroidetes*, that might specifically produce CD8+ T cells unique to the microbiota having anticancer capabilities (Tanoue et al., 2019). Moreover, *Bacteroidetes* species produces integrase that contains a motif which raised the risk of systemic damage to the pancreas in Type 1 diabetes mellitus (T1DM) while simultaneously increasing CD8+ T cells in the gut (Nanjundappa et al., 2017). In a mice model with altered gut microbiota enriched in *Fusobacteria* expressed a magnesium transporter (Mgt), that accelerated the development of diabetes. An islet-specific glucose-6-phosphatase catalytic subunit–related protein (IGRP)-mimicking peptide carried by this transporter stimulated the CD8+ T cells specific to IGRP and caused diabetes in vivo (Tai et al., 2016).

Studies have suggested that the major SCFAs, like acetate, butyrate and propionate, which are metabolites of microbes, are involved in the mediation of CD8+ T cell function. A study demonstrated increased acetate levels in MS patients' blood, which were associated with CD8+ T cells that produce IL-17 (Pérez-Pérez et al., 2020). It is worth noting, the diet high in acetate and lesser butyrate level in non-obese diabetic (NOD) mice model of T1DM decreased the frequency of autoreactive CD8+ T cells and increased the number of CD4+Foxp3+ Treg cells in the spleen and colon, but there was no change in the peripheral lymph nodes (Mariño et al., 2017). Following this diet plan might decrease the frequency of pathogenic autoreactive T cells in MS and T1DM by shifting the balance toward a more regulatory T cell profile, thereby potentially mitigating disease progression. Through APCs, butyrate and propionate regulate the activation of CD8+ T cell and IL-12 production (Nastasi et al., 2017). According to another study, butyrate directly boosts the activity of CD8+ T cell by enhancing the expression of granzyme B and IFNγ (Luu et al., 2018). Thus, microbiome-immune axis could reveal new adjuvants for the treatment of certain AIDs, tailored to individual microbiota profiles.

IFNγ based CD8+ T cell induction might be linked with particular metabolites produced by bacterial strains, such as dimethylglycine, mevalonate and SCFAs (Tanoue et al., 2019; Bachem et al., 2019), which enter the bloodstream and cause CD8+ T cell systemic activation. As a result, the microbiota can influence the effectiveness of immunotherapy and CD8+ T cell function. Butyrate from *Lachnospiraceae* species was found to inhibit CD8+ T cells that secreted IFNγ. Butyrate inhibited DCs based stimulation of the IFN gene (STING), that is linked to responses of CD8+ T cell and reduces the effectiveness of radiation therapy (Yang et al., 2021). Pentanoate produced by *Megasphaera malasiliensis* stimulates the activity of effector CD8+ T cells. Higher levels of TNFα and IFNγ were found in the presence of *M. massiliensis*, and this had a favorable impact on adoptive T cell therapy's effectiveness (Luu et al., 2021). Future studies should focus on enhancing the efficacy of adoptive T cell therapies for controlling CD8+ T cell responses that may be done by engineering microbes to produce beneficial metabolites, such as acetate, butyrate and pentanoate.

### Natural killer T cells (NKT)

NKT cells, resembles conventional T cells and can be influenced by the microbiota and have a role in autoimmune diseases (Simoni et al., 2013). NKT cells can sense the surrounding lipid environment by engaging their TCR with CD1-expressing cells, as opposed to CD4+ T cells' ability to sense protein antigens (Cheng et al., 2024). According to studies, gram-negative bacteria like *Sphingomonas* act as NKT cell stimulators, and commensal microbiota regulate the homeostasis of NKT cells. As microbial antigens, the glycosphingolipids and glycosylceramides derived from *Sphingomonas* induce NKT cell activation and secretion of IFNγ (Kinjo et al., 2005; Mattner et al., 2005). Moreover, 40–70% of the membrane phospholipids in the *Bacteroides* genus contain sphingolipids, which CD1d may present to NKT cells, the bacteria of this genus may be essential for controlling the population of NKT cells. *B. fragilis* synthesize an isoform of αGalCer. In a CD1d-dependent manner, this sphingolipid has been demonstrated to activate and stimulate the production of IFN-γ in both human and mouse NKT cells (Brown et al., 2021; Brailey et al., 2020). This approach may improve pathogen clearance and immune surveillance in chronic infections like cancer, with dosage and glycosphingolipid combinations personalized based on individual microbiome profiles.

A study reported that the commensal bacteria decreases the accumulation of mucosal NKT cells. The frequency and quantity of colon NKT cells were found to be higher in germfree mice compared to SPF mice, indicating a possible link between allergic asthma morbidity and IBD (Olszak et al., 2012). In neonatal mice, sphingolipid generated by *B. fragilis* suppresses NKT cell proliferation, regulating homeostasis (An et al., 2014). Pneumolysoid and type-3-polysaccharide (T3P) produced by *Streptococcus pneumoniae* stimulate Tregs, which reduce airway hyperreactivity dependent on NKT cell (Thorburn et al., 2012). Microbial bile acids regulate the accumulation of hepatic NKT cell through mediating CXCL16 (CXC-motif ligand 16) expression. Liver sinusoidal endothelial cells express more CXCL16 when exposed to bile acids modified by *Clostridium* species. Additionally, hepatic NKT cell recruitment has demonstrated remarkable anti-tumor responses against tumors of EL4 lymphoma (Ma et al., 2018). Therefore, it will be crucial to look into whether changes in microbiome of AIDs patients influence the pathophysiology of the disease by regulating NKT cells.

### γ*δ* T cells

γδ T cells are known to have semi-invariant TCRs consisting of γ and δ chains, and they are connected to many homeostatic functions such as wound healing and immunological monitoring (Ribot et al., 2021). In various disease models, γδ T cells play an important role as early responders, and frequently they produce the first cytokines like IFN-γ and IL-17 (Ribot et al., 2021; Chien et al., 2014). Although the amount of intestinal γδ intraepithelial lymphocytes (IELs) appears to be unaffected by the gut microbiota, some commensals can raise the frequency of γδ T cells that are positive for IL-17 and IL-1R1 by signaling via VAV1 (a guanine nucleotide exchange factor), which may provide protection against illness (Duan et al., 2010). Thus, there is need to investigate the type of commensals that increases IL-17 and IL-1R1-positive γδ T cells as it may result in the development of innovative probiotics for immune homeostasis. Moreover, the homeostasis of γδ T cells, which produce IL-17A, is also facilitated in the liver by the commensal microbiota. This disturbs the activation of these cells and their secretion of IL-17 cytokines, which in turn affects the advancement of NAFLD (Li et al., 2017a).

Li et al. (2017b) findings showed that via dose-dependent manner hepatic γδ T cell restoration was done by *Escherichia coli*. Additionally, immune surveillance was restored in mice treated with antibiotics when IL-17A and γδ T cells were added (Cheng et al., 2014). A study determined that microbial metabolite, propionate works as a major component in regulating γδ T cells based release of IL-17 (Dupraz et al., 2021). In the colon, *Lactobacillus breves* DM9218 expressed TLR2, which directly stimulated γδ T cells and had positive effects on colitis (Li et al., 2018). Certain beneficial bacteria, like *Bacillus spp.* and *Bifidobacterium*, increased TLR2 expression, which improved barrier functions via γδ T cells (Li et al., 2017b). Indeed, certain gut commensals can enhance IL-17 and IL-1R1-positive γδ T cells, that suggests using targeted probiotics might enhance immune defense. More specific insights related to these strains can lead to novel treatments for conditions like NAFLD and colitis by upregulating beneficial T cell activity. Table 2 presents the types of microbiota or metabolites involved in the manipulation of T cell subtypes functions and their effects.

**Table 2:** Gut microbiota/metabolites mediated manipulation of T cell subtypes function and their outcomes.

T cell subtype	Types of microbes/metabolites	Impact on immune system regulation
CD4+ T cells	*Klebsiella pneumonia*, *Klebsiella aeromobilis* (Atarashi et al., 2017)	Induce Th1 cell proliferation, by increasing their numbers
	*Lactobacillus salivarius* (Ren et al., 2019), *Lactiplantibacillus plantarum* (Matsusaki et al., 2016; Takeda et al., 2011) *Lacticaseibacillus casei* (So et al., 2008)	Increase Th1 cytokines (TNFα, IFNγ), regulate Th1 and Th2 responses Inhibit the Th1 cells and enhance the antigen-specific oral tolerance
	*Bacteroides fragilis* (PSA) (Mazmanian et al., 2005)	Balance Th1/Th2 responses, correct Th2 dominance in germ-free mice
	*Lachnospiraceae* (A4 bacteria) (Wu et al., 2016)	Produce TGF-β, prevent Th2 cell differentiation and activity
	Gram-positive bacteria, SFB (Ivanov et al., 2008) *Porphyromonas gingivalis* (Moen et al., 2006)	Induce Th17 cell differentiation, promote IL-17 and IL-22 production Activate Th17 pathway activation via systemic proinflammatory cytokine profiles
	*Clostridium* spp.	Influence Treg cell differentiation and function, impact immune tolerance
	*Bifidobacterium* (Sun et al., 2020)	Promote Treg cell development by encouraging their mitochondrial activity
	*Lactobacillus reuteri* (Forsythe et al., 2007), *Lactobacillus acidophilus* (L-92) (Shah et al., 2012), *Lactobacillus murinus* (Bernard-Raichon et al., 2021)	Increase Foxp3 expression, promote Treg cell development, reduce pulmonary inflammation
	*Bacteroides fragilis* (Round and Mazmanian, 2010) *Monoglobus, Megamonas* and *Prevotella* (Wang et al., 2022b)	Generate large populations of Treg cells via PSA production Favorably affect the Th17/Treg ratio and Treg cell abundance, which may reduce RA disease activity. They also positively influence cytokine levels and CD4+ T cell numbers.
	Vancomycin (Gram-positive bacteria antibiotic) (Ivanov et al., 2008)	Decrease Th17 cell occurrence in small intestine, indicating role in Th17 differentiation
CD8+ T cells	*Ruminococcus* species (Shimokawa et al., 2020) SCFAs (Acetate) (Pérez-Pérez et al., 2020)	Stimulate CD8+ T cells. Increased acetate levels in MS patients’ blood associated with CD8+ T cells producing IL-17.
	SCFAs (Butyrate, Propionate) (Nastasi et al., 2017),(Luu et al., 2018)	Regulate CD8+ T cell activation and IL-12 production via APCs, enhance granzyme B and IFNγ expression
	*Megasphaera massiliensis* (Pentanoate) (Luu et al., 2021)	Stimulate effector CD8+ T cells, increase TNFα and IFNγ levels
	*Lachnospiraceae* species (Butyrate) (Yang et al., 2021)	Inhibit CD8+ T cells secreting IFNγ, reduce effectiveness of radiation therapy via inhibition of DCs’ STING
	Dimethylglycine, Mevalonate, SCFAs (Tanoue et al., 2019, Bachem et al., 2019)	Induce systemic CD8+ T cell activation, enhancing immunotherapy effectiveness
	NKT cells	*Sphingomonas* (Kinjo et al., 2005, Mattner et al., 2005)*Bacteroides fragilis* (Sphingolipid) (An et al., 2014) *Streptococcus pneumonia* (Thorburn et al., 2012)	Act as NKT cell stimulators, induce IFNγ secretion Suppress NKT cell proliferation in neonatal mice, regulate homeostasis Produces pneumolysoid and type-3-polysaccharide (T3P) which stimulate Tregs and reduce airway hyperreactivity dependent on NKT cell	
*Clostridium* spp. (Bile Acids)		Regulate hepatic NKT cell accumulation via CXCL16 expression, demonstrate anti-tumor responses
γδ T Cells			Commensal microbiota (Li et al., 2017b) Propionate (Dupraz et al., 2021) *Lactobacillus breves* DM9218 (Li et al., 2018) *Bacillus* spp. and *Bifidobacterium* (Li et al., 2017b)	Influence IL-1R1 expression, sustain IL-17 production, regulate homeostasis of γδ T17 cells. Regulate γδ T cell motility and localization in the gut epithelial layer, essential for gut immunosurveillance. Regulate IL-17 release by γδ T cells Express TLR2, directly stimulate γδ T cells, positively affect colitis. Increase TLR2 expression, improve barrier functions via γδ T cells.

## Disease specific composition and functionality of different gut microbial species in major AIDs

**Figure 5:**
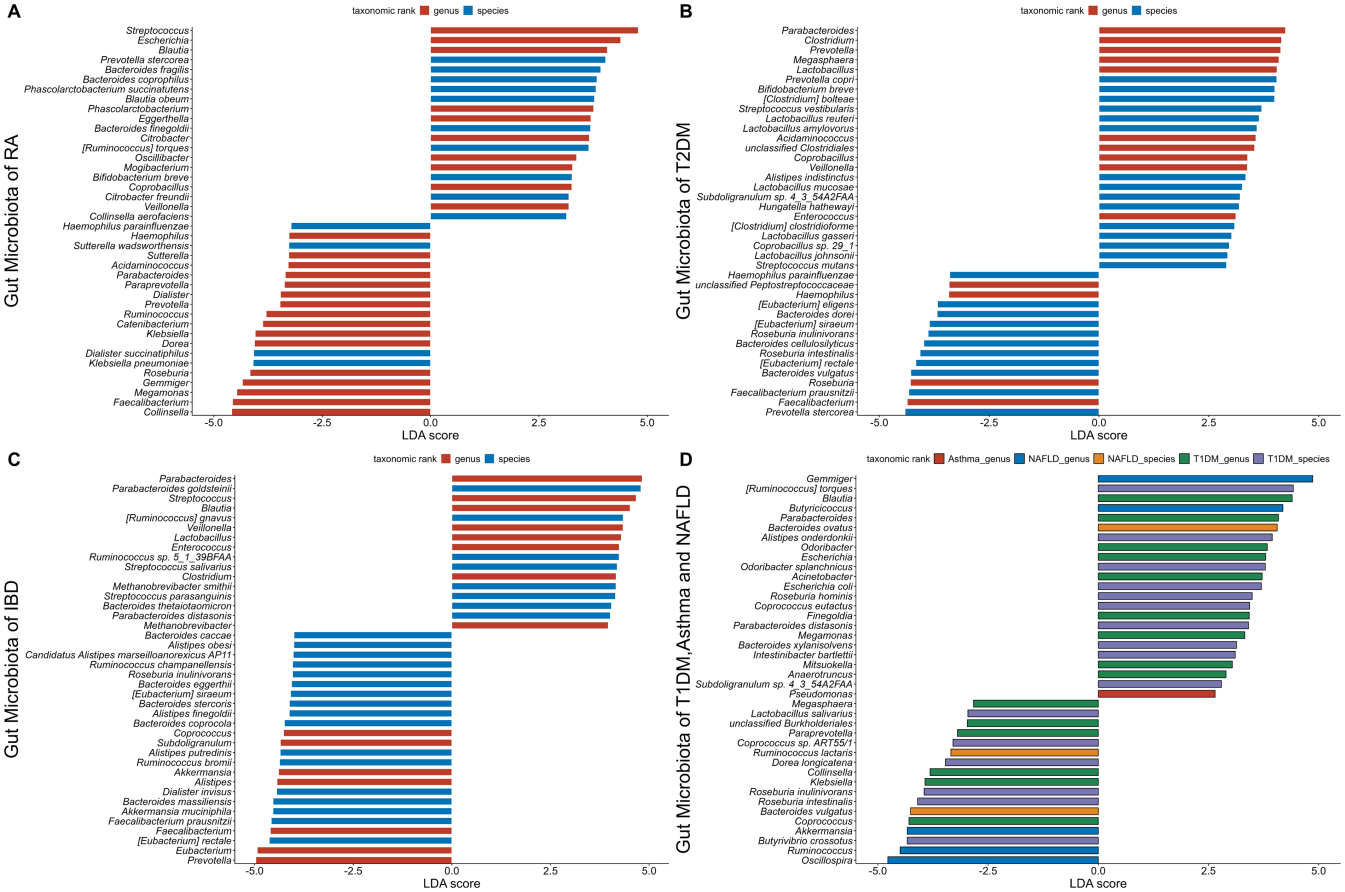
The gut microbiota associated with six autoimmune diseases (RA, T1DM, T2DM, Asthma, IBD, NAFLD). The LDA scores less than 0 indicate Health enriched taxa, while LDA scores larger than 0 indicate autoimmune diseases enriched taxa. The length of the bar chart represents the significance of differential phenotypes. **(A–C)** show the top 40 gut microbiota with the highest LDA scores for RA, T2DM, and IBD, respectively. **(D)** shows the gut microbiota with LDA scores greater than 0 for T1DM, asthma, and NAFLD. Nr. samples: In GMrepo, manually curation was performed for selected NCBI BioProjects in order to group samples according to their corresponding phenotypes to obtain Nr. samples, and identify marker taxa between a pair of phenotypes of interests, e.g., RA vs. Health. The total Nr. sample size involved in figure is Rheumatoid arthritis (RA,N = 233;HC,N = 174), Type 1 diabetes mellitus (T1DM,N = 113;HC,N = 153), Type 2 diabetes mellitus (T2DM,N = 240;HC,N = 264), Asthma (asthma,N = 145;HC,N = 1,451), Inflammatory bowel disease (IBD,N = 241;HC,N = 126), Non-alcoholic fatty liver disease (NAFLD,N = 81;HC,N = 62).

Environmental factors and genetic predispositions are both known to enhance the development of AIDs, along with a disturbed gut microbiota emerging as a major area of interest. Research has indicated that several AIDs are influenced with deviations in the gut microbiota's composition and functionality. There is mounting evidence to suggest that the immunopathogenesis of AIDs is influenced by these disruptions in the gut microbiota. In the GMrepo database, we compared healthy phenotypes with six AIDs phenotypes (RA, T1DM, T2DM, Asthma, IBD, NAFLD) and identified gut microbiota significantly associated with the six AIDs, characterized by LDA scores. The higher the score, the more significant the relationship between gut microbiota and certain AIDs (Figure 5) (Wu et al., 2020).

### Rheumatoid arthritis (RA)

RA is a type of chronic AID that destroys joints and impairs its function. Recently, certain genetic factors along with gut dysbiosis has been proposed as the pathogenesis of RA (Maeda et al., 2016; Jubair et al., 2018). Microbiome research on germfree mice highlights the importance of instability in gut microbiota composition for the etiology of arthritis (Scher et al., 2013). Patients having RA exhibit markedly altered compositions of gut microbiota, with a decrease in *Faecalibacterium*, a beneficial microbe, and an increase in *Prevotella* species such as *Prevotella copri* (Wells et al., 2020). In a mouse model of arthritis, oral exposure to *Prevotella nigrescens* and *Porphyromonas gingivalis* exacerbated the inflammation by inducing IL-17 production by the immune system and promoting a Th17 cell response (de Aquino et al., 2014). In addition, *Collinsella* has been linked to cause severe arthritis in mouse models by promoting the expression of IL-17A and thereby increasing gut permeability. *Collinsella* has been found to be elevated in RA patients (Chen et al., 2016a). In early stages of RA, the patients have a dominant gut microbiota that includes *Collinsella* (Koh et al., 2023) and *Prevotella copri* (Zhang et al., 2015), indicating their potential roles in the etiology of disease. *P. gingivalis* has been found in the serum of RA patients as well as those who are at verge of developing RA (Mikuls et al., 2012).

The link between RA and SCFAs in the diet has been brought to light by many researches. Because of their function as histone deacetylase inhibitors, SCFAs especially butyrate, suppress the inflammation in RA (Chen et al., 2016b; Maslowski et al., 2009). Research has demonstrated that butyrate inhibits RA by suppressing the inflammatory cytokines secretion and by regulating Treg cells (Takahashi et al., 2020; Yang et al., 2024). By blocking histone deacetylase (HDAC)2 activity in osteoclasts and HDAC8 activity in T cells, butyrate decreased the inflammation in a CIA mice model, which in turn decreased the joint inflammation (Kim et al., 2018). In addition, in RA models, mice lacking SCFA receptors show increased inflammation (Maslowski et al., 2009). The influence of gut microbiota on RA is indicated by the presence of species such as *Porphyromonas* and the decrease in beneficial microbe like *Faecalibacterium*. Additionally, SCFAs such as butyrate have anti-inflammatory properties; this implies that dietary changes may help control RA by influencing immunological responses and gut health.

### Type 1 diabetes mellitus (T1DM)

T1DM is a type of AIDs, caused when T lymphocytes attacks and destroys insulin-producing β-cells of pancreas. The link between T1DM and the gut microbiota is evident by the reduced numbers of intestinal Treg cells along with healthy microbiota in individuals suffering with T1DM (Anderson, 2023). Studies conducted on humans from a variety of ethnic backgrounds have consistently reported changes in gut microbiota in relation to T1DM (Kostic et al., 2015; Needell and Zipris, 2016; Mejía-León et al., 2014), marked by an increase in *Bacteroides* species and a decrease in the amount of bacteria that produce SCFA, namely *F. prausnitzii* (de Goffau et al., 2014; de Goffau et al., 2013; Chukhlovin et al., 2023). Children with T1DM, had lower levels of members of *Clostridium* clusters IV and XIVa (de Goffau et al., 2013). Moreover, the individuals with T1DM have been found to have decreased expression of intestinal FOXP3, an important transcription factor for the Treg cells activation (Badami et al., 2011). Additional data suggests that microbial diversity was lower (Kostic et al., 2015) and intestinal permeability was higher (Maffeis et al., 2016) prior to the T1DM diagnosis.

In a recent research, when LEfSe analysis was performed, 28 bacterial taxonomic clades showed statistically major alterations (13 elevated and 15 reduced) in T1DM patients in comparison to healthy controls. *Porphyromonadaceae*, a family of the *Bacteroidetes* phylum, was overrepresented in T1DM patients, but *Paenibacillaceae*, *Veillonellaceae*, *Ruminococcaceae*, and *Phascolarctobacterium*, all belonging to the *Firmicutes*, were most abundant in healthy individuals. Furthermore, the phylum *Fusobacteria* was differentially enriched in healthy participants (Abuqwider et al., 2023).

Studies on animals using NOD mouse models have clarified the SCFAs protective applications against T1DM. For instance, NOD mice of T1DM given specific diets that increased the amount of acetate and butyrate released by the bacteria which resulted in higher number of Treg cells and lower number of autoreactive T cells and protected them against the development of T1DM (Mariño et al., 2017). Furthermore, in a recent study conducted on T1DM model of NOD mice reported, decrease in the number of SCFAs (Wang et al., 2023b). Interestingly, gut permeability is shown to be a critical mediator between intestinal microbiota, inflammation, and the onset of T1DM, with implications for both human and animal models (Vaarala et al., 2008). Therefore, it is logical to hypothesize that SCFAs could preserve the integrity of the gut barrier by modifying the gut microbiota, encouraging tight junctions and thickening of the mucus layer, which would prevent the onset of T1DM.

### Type 2 diabetes mellitus (T2DM)

The symptoms of T2DM include insufficient insulin secretion, increased insulin sensitivity and hepatic glucose production. According to recent studies, dysbiosis may have played a role in the development of T2DM (Arora et al., 2021). Numerous studies examining the gut microbiota of T2DM patients have discovered significant genus-level variations between the patients and healthy controls. These results suggest a connection between diabetes and modifications in the gut microbiota. Frequently reported results indicate that *Ruminococcus, Fusobacterium,* and *Blautia* have positive associations with T2DM, while the genera *Akkermansia, Bacteroides, Bifidobacterium, Faecalibacterium* and *Roseburia* have negative associations (Larsen et al., 2010; Gurung et al., 2020).

In comparison to healthy controls, the gut microbiota of individuals having T2DM from Northern China had lower levels of *Akkermansia* and *Bifidobacteria* species, but higher levels of *Dorea* (Li et al., 2020). In a metagenome-wide association study, adequate level of gut dysbiosis was found in patients having T2DM. Control samples had higher levels of *Lactobacillus spp.* and butyrate-producing bacteria, T2DM patients had higher levels of opportunistic pathogens like *Clostridium spp.* (Qin et al., 2012). Reduced levels of butyrate producing bacteria, which affect insulin sensitivity, are associated with T2DM (Vrieze et al., 2014). A diet that produces acetate and butyrate has been shown to improve gut integrity, stimulate Treg cells, secrete IL-21, and prevent T2DM (Puddu et al., 2014). Through particular G protein receptors (GPR41, GPR43), SCFAs cause intestinal L-cells to secrete GLP-1, which affects insulin release, pancreatic function, and central effects on appetite regulation (Fava, 2014).

In addition to SCFAs, gut microbiome alpha diversity has been linked to other serum metabolites. A microbiome-metabolite score that combined the levels of these metabolites in circulation showed stronger correlations with cardiometabolic traits. Significantly, this score was connected to the incidence and prevalence of T2DM, indicating that metabolites derived from the microbiome play a mechanistic role in the relationship between the composition of the microbiome and health (Menni et al., 2020). While considering the link between dysbiosis of the gut microbiota and T2DM, alteration of gut microbiota may prove to be a useful therapeutic approach. Insulin sensitivity and secretion may be improved by the development of personalized probiotic therapy to increase SCFA-producing bacteria. Moreover, a combined microbiome-metabolite score may also be utilized to mitigate the onset and progression of T2DM by enabling early diagnosis.

### Atopic asthma

Reduced metabolic capacity and early-life dysbiosis of the gut microbiota can impair pulmonary and local immunity, making individuals more vulnerable to lung disorders like atopic asthma (Zhao et al., 2023). Microbes in the environment may have an impact on asthma risk even before birth. Numerous studies have demonstrated that exposure of mother to a farming environment protects her offspring from allergic reactions, such as asthma (Roduit et al., 2011; Loss et al., 2012; Ver Heul et al., 2019). Animal models used in experiments, especially newborn mice given antibiotics, showed reduced diversity of gut microbes, altered metabolite profiles, elevated response of immune cells and greater vulnerability to allergic inflammation of lungs (Russell et al., 2012; Cait et al., 2018). Moreover, the inflammation was reduced in these mice models when SCFAs were added in their diet. The mechanism for this improvement was linked to lower levels of circulating IgE and immune--modulating markers like T cells and IL-4-producing CD4+ T cells (Cait et al., 2018).

The relationship between the gut microbiota and asthma was investigated in a clinical research involving 58 patients having asthma and healthy controls. The findings showed a favorable relationship of the relative abundance of *Bifidobacterium* and *Lachnospiraceae* family with asthma. On the other hand, asthmatic participants had a decrease in the relative abundance of *Bacteroides* and *Enterobacteriaceae* family (Begley et al., 2018). Moreover, *Proteobacteria* was found to be the predominant phylum overrepresented in human observational studies (Wang et al., 2022a), which connected pro-inflammatory mechanisms to the pathogenesis and severity of asthma. *Bifidobacterium* administration has been demonstrated to increase IL-10–producing Treg cells, that aid in suppressing over reactive immune responses. Individuals with asthma who received a *Bifidobacterium* mixture in a randomized controlled experiment, reported better quality of life and clinical symptoms than those who received a placebo (Miraglia Del Giudice et al., 2017).

In human airway inflammation, SCFAs, known to be protective bacterial metabolites showed anti-inflammatory effects. The two most common bacterial phyla that produce SCFAs are *Firmicutes* and *Bacteroidetes,* and both are capable of producing acetate. In *Firmicutes*, (*Coprococcus*, *Clostridium*, and *Ruminococcus*) are the primary producers of butyrate. *Bacteroidetes* like *Prevotella* produce propionate (Zhao et al., 2023). Soluble fiber demonstrated anti-inflammatory properties through the binding of SCFAs to related G-protein-coupled receptors (GPCRs) (McLoughlin et al., 2019). Research also indicates the production of pro- and anti-inflammatory metabolites like histamine (Pugin et al., 2017) and oxylipins like 12,13-diHOME (Stewart, 2019) by gut bacteria, pointing a complex interaction between gut microbiota and asthma. Dietary fiber can be used by gut microbes to produce SCFAs, in order to control immunological response of the host (Silva and Bernardi, 2020). By modifying the development and activity of immune cells, SCFAs may be crucial in controlling asthma (Niu et al., 2023). The associated mechanisms that facilitate networking between the lungs and gut are still uncertain. More specifically, the pro-inflammatory responses in the lungs are inhibited by SCFAs derived from gut bacteria. Therefore, targeted focus on fostering SCFA-producing bacteria like *Bifidobacterium* and *Prevotella* might be a potential way to decrease asthma risk by promoting anti-inflammatory effects in the respiratory tract.

### Inflammatory bowel disease (IBD)

IBD is a multifactorial disease, caused by environmental factors, genetic factors, and immune-mediated factors caused by microbiota and it consists of Crohn's disease (CD) and ulcerative colitis (UC) (Diez-Martin et al., 2024). Rather than a single causative organism, several microbes dysbiosis is linked to the onset of IBD (Lepage et al., 2011). Several studies show that gut microbes, which differ in composition and functionality from healthy controls, play a critical role in the manifestation of IBD (Thursby and Juge, 2017; Pandey et al., 2024). *B. longum* Bar 33 and *L. acidophilus* Bar 13 decrease the quantity of intraepithelial lymphocytes and increase the growth of Treg cells in mice models of intestinal inflammation caused by 2,4,6-trinitrobenzene sulphonic acid-induced colitis (Roselli et al., 2009). By stimulating and growing colonic CD4+ FoxP3+ Treg cells, *L. casei* DN-114 001 reduces the severity of the disease in a mouse colitis paradigm (2,4-dinitrobenzene sulphonic acid) (Hacini-Rachinel et al., 2009). Recently, a study on mice colonized with *B. fragilis* and a healthy human microbiota, highlighted that colitis induction resulted in a decrease in PSA in the "ON" orientation, which was reversed when inflammation decreased (Carasso et al., 2024). Similarly, a study employing colitis-prone mice shown that *Enterobacter ludwigii* was successful in reducing colitis symptoms. When compared to other antibiotic treatments, metronidazole had the greatest effect in reducing colitis in a colitis prone mouse model caused by dextran sulfate sodium (DSS). This effect was linked to an increase in the abundance of the gut microbiota species *E. ludwigii*. By using metabolites from *E. ludwigii* to stimulate Treg cells differentiation, the immunological tolerance response was boosted and mice's susceptibility to DSS-induced colitis was decreased (Li et al., 2022a).

Reduced microbial diversity, particularly a decline in *Firmicutes* and an up rise in *Proteobacteria* taxa, is indicative of IBD-associated dysbiosis. Reductions in the number of *Firmicutes* bacteria from the *Lachnospiraceae* and *Ruminococcaceae* families (Matsuoka and Kanai, 2015; Halfvarson et al., 2017), which are essential for the synthesis of butyrate, are frequently observed in active CD. Decreased butyrate production capacity is one of the functional disturbances associated with this depletion (Marchesi et al., 2007). Researches have reported reduced abundance of *Blautia faecis*, *Clostridium lavalense*, *Roseburia inulinivorans and Ruminococcus torques* in individuals having CD (Takahashi et al., 2016; Pandey et al., 2024). Findings in the ileal CD microbiota showed a reduced number of genes involved in the SCFAs synthesis, along with a decline in the butyrate-producing bacteria *F. prausnitzii* and *Roseburia sp.* (Liu et al., 2021).

Butyrate is known to treat IBD due to its ability to support colonocyte energy, inhibiting inflammation and improving the integrity of the epithelial barrier. A few studies applied a different strategy that uses probiotics to uplift in situ butyrate production by consuming butyrate-producing bacteria (Miquel et al., 2013; Tamanai-Shacoori et al., 2017). In a recent study, mice were protected against sorbitol-induced diarrhea by inoculation with butyrate-producing *Anaerostipes caccae*, even after the elimination of probiotic, which returned the abundance of *Clostridia* back to normal (Lee et al., 2024). This strategy suggests that focusing on microbial dysbiosis through supplementation of bacterial species which produce butyrate may be helpful in reestablishing the gut homeostasis and improving health of IBD patients.

### Non-alcoholic fatty liver disease (NAFLD)

A rising global health concern, NAFLD is associated with an increased risk of cancer and liver disease. In this disease, there is excessive buildup of fat in the liver which is not linked with consumption of alcohol (Tang et al., 2024). Through a variety of immunological, epigenetic and metabolic processes, intestinal dysbiosis is a major contributor of NAFLD (Chen et al., 2020; Wolfe et al., 2023). The gut microbiota profile in individuals having NAFLD is strongly associated with systemic inflammation and can coexist with the development of hepatocellular carcinoma (Ponziani et al., 2019). Research indicates that in both NAFLD and non-alcoholic steatohepatitis (NASH), there is a reduction in alpha and beta diversities accompanied by modified microbial signatures (Caussy et al., 2019). These signatures include a rise in *Enterobacteriaceae, Proteobacteria* and genera like *Collinsella, Dorea, Escherichia,* and a decline in *Coprococcus, Faecalibacterium, Eubacterium* and *Prevotella* (Hoyles et al., 2018; Boursier et al., 2016). Despite the fact that these preliminary findings point to a detectable difference in microbial profiles between hepatic steatosis patients and controls, significant differences are recorded between studies with opposing findings in the literature. *P. copri* was found to be linked with a higher progression risk in both NAFLD and NASH, that may have connection with reduced capacity of butyrate production and elevated intestinal permeability (Moran-Ramos et al., 2023). Moreover, in the NASH, there is a reduction in the levels of the butyrate producer *F. prausnitzii*, a common microbial signature linked to other metabolic diseases (Caussy et al., 2019; Iebba et al., 2018).

Furthermore, it is possible that the increased *Lactobacillus* presence increases the amount of SCFAs in the gut, which have been demonstrated to reduce fat accumulation (Hou et al., 2022). According to another study, yinchen linggui zhugan decoction (YLZD) improved NAFLD treatment and raised *Christensenellaceae* abundance (Jiang et al., 2022). As for now, it is unknown how *Christensenellaceae* affects NAFLD. It is possible therefore, that this association is related to the improvement of fat metabolism (Tavella et al., 2021). There is a documented inverse relationship between insulin resistance and *Christensenellaceae* abundance. There may be a connection between *Christensenellaceae* and the onset of NAFLD because lower quantity of this bacterium is linked to more severe insulin resistance and consequent fat storage (Chen et al., 2021b). Through different mechanisms, the gut microbiota is linked to the onset and progression of NAFLD. When bacteria, like *Collinsella sp.* metabolize bile acids into oxo-bile acid intermediates, it can lead to increased intestinal permeability, which can exacerbate NAFLD (Doden et al., 2018; Purohit et al., 2024).

A large number of toxic metabolites that cause liver fibrosis and inflammation may be accessible to the liver due to dysbiosis and increased intestinal permeability. To shield liver cells from harmful or toxic metabolites the gut mucosal barriers must remain intact (Park et al., 2022). *A. muciniphila* not only improves the gut barrier integrity but also boosts Treg cell numbers and inhibits pro-inflammatory Th17 responses, both of which can help to prevent or lessen NAFLD condition (Nian et al., 2023). It is interesting to note that although butyrate and propionate were more common in mild to moderate NAFLD, the SCFAs especially acetate, were enriched in advanced NAFLD stages, suggesting different roles in disease severity (Aoki et al., 2021). Thus, the modulation of NAFLD progression is influenced by specific microbial metabolites that alter host metabolic and immune pathways. Moreover, more production of oxo-bile acids and SCFAs, by bacteria such as *Collinsella sp.* and *Lactobacillus* impact intestinal permeability and systemic inflammation. Targeting these microbial metabolic pathways could provide novel therapeutic strategies and personalized medicine methods for the control and therapy of NAFLD. Table 3 highlights the associations between the changes in gut microbiota, the role of SCFAs, and the mechanisms that leads to the development and progression of each autoimmune disease mentioned in this section.

**Table 3:** The associations between gut microbiota changes, the role of SCFAs, and the mechanisms contributing to the development each autoimmune disease.

Autoimmune Diseases	Key microbiota changes	SCFA role and impact	Relevant mechanisms
Rheumatoid Arthritis (RA)	↑ *Prevotella copri* (Wells et al., 2020), *Collinsella* (Chen et al., 2016a) *Porphyromonas gingivalis* and *Porphyromonas nigrescens* (de Aquino et al., 2014) ↓ *Faecalibacterium* (Wells et al., 2020)	SCFAs, especially butyrate, suppress RA inflammation	Altered gut microbiota linked to increased gut permeability, dysbiosis, and elevated zonulin levels contributing to joint inflammation
Type 1 Diabetes Mellitus (T1DM)	↑ *Bacteroides* (de Goffau et al., 2014; de Goffau et al., 2013; Chukhlovin et al., 2023), *Porphyromonadaceae* (Abuqwider et al., 2023) ↓ SCFA-producing bacteria (e.g., *Faecalibacterium prausnitzii*) (de Goffau et al., 2014, de Goffau et al., 2013, Chukhlovin et al., 2023), *Paenibacillaceae*, *Veillonellaceae*, *Ruminococcaceae*, *Phascolarctobacterium* and *Fusobacteria* (Abuqwider et al., 2023)	SCFAs protect against T1DM, increased SCFA intake reduces T1DM incidence in NOD mice	Gut permeability, decreased microbial diversity and lower SCFA biosynthesis genes linked to T1DM onset
Type 2 Diabetes Mellitus (T2DM)	↑ *Ruminococcus, Fusobacterium, Blautia* (Larsen et al., 2010; Gurung et al., 2020), *Dorea* (Li et al., 2020), *Clostridium* spp. (Qin et al., 2012) ↓ *Akkermansia, Bacteroides* (Li et al., 2020)*, Bifidobacterium, Faecalibacterium, Roseburia* (Larsen et al., 2010, Gurung et al., 2020)	Reduced SCFAs production affects insulin sensitivity, SCFAs via GPRs stimulate GLP-1 secretion	Gut dysbiosis, reduced butyrate production, gut microbiome diversity linked to T2DM development
Atopic Asthma	↑ *Bifidobacterium*, *Lachnospiraceae* (Begley et al., 2018) and *Proteobacteria* (Wang et al., 2022a) ↓ *Bacteroides* and *Enterobacteriaceae* (Begley et al., 2018)	SCFAs have anti-inflammatory effects, reduce airway inflammation	Early-life exposures affect microbiota, SCFAs reduce lung inflammation via immune modulation
Inflammatory Bowel Disease (IBD)	↑ *Proteobacteria* (Matsuoka and Kanai, 2015; Halfvarson et al., 2017) ↓ *Firmicutes* (Matsuoka and Kanai, 2015, Halfvarson et al., 2017), *Blautia faecis*, *Clostridium lavalense*, *Roseburia inulinivorans and Ruminococcus torques* (Takahashi et al., 2016, Pandey et al., 2024), butyrate-producing bacteria (e.g., *Faecalibacterium prausnitzii*) and *Roseburia* sp. (Liu et al., 2021)	Butyrate supports colonocyte energy, inhibits inflammation	Microbial dysbiosis, decreased SCFA synthesis genes, use of probiotics for butyrate production to treat IBD
Non-Alcoholic Fatty Liver Disease (NALFD)	↑ *Enterobacteriaceae, Proteobacteria, Collinsella, Dorea, Escherichia* (Hoyles et al., 2018; Boursier et al., 2016) and *Prevotella copri* (Moran-Ramos et al., 2023) ↓ *Coprococcus, Faecalibacterium, Eubacterium, Prevotella* (Hoyles et al., 2018, Boursier et al., 2016)	SCFAs reduce fat accumulation, role varies by disease stage (butyrate/propionate vs. acetate)	Gut-liver axis, increased permeability and endotoxemia, microbial metabolites affect liver inflammation

**↑: increased; ↓: decreased levels of certain microbial populations.**

## Conclusion and future perspectives

The etiology of autoimmune disorders is linked to gut dysbiosis. Therefore, in order to gain a deeper understanding of the effects of gut dysbiosis, we found the altered gut bacteria that were common in AIDs mentioned in this article and what effects they and their metabolites bring on immune system. It is interesting to note that the production of autoantibodies or the Th17 cells activation in reports of immune-related diseases are partly correlated with the common, changed gut bacteria that are abundant in AIDs. Notably, Beneficial gut microbiota strains like *F. prausnitzii*, *A. muciniphila*, and *Roseburia sp.* secrete immunomodulatory metabolites such as SCFAs, which support Treg cells production, while suppressing the inflammatory pathways in AIDs such as SLE, pSS, SpA, AS, Lupus nephritis, RA, IBD, T1DM, T2DM, Atopic asthma and NAFLD. Conversely, pathogenic strains, including *P. copri* and *R. gnavus*, disrupt gut barrier integrity by promoting pro-inflammatory Th17 polarization, and exacerbate immune dysregulation, contributing to disease progression.

The gut microbiota alterations have disease-specific implications. For example, in RA disease increased *P. copri* and decreased SCFA-producing bacteria correlate with heightened inflammation and disease severity. In SLE, elevated *R. gnavus* levels are associated with lupus nephritis, whereas beneficial species like *Bacteroides sp*. are depleted. IBD is marked by a loss of butyrate-producing bacteria like Roseburia and overrepresentation of pro-inflammatory *Proteobacteria*, contributing to chronic intestinal inflammation. It is acknowledged that different strains within the same species might differ significantly in terms of their pathogenicity and functionality (Conway and Cohen, 2015). For example, *B. fragilis* strains that are enterotoxigenic can stimulate Th17 responses through their enterotoxin (Wick et al., 2014), while non-toxigenic strains can trigger Treg cell responses through their capsular polysaccharide A (Wang et al., 2014). Therefore, it is worth understanding that the relationship between the gut microbiota and host immune system is so complicated and understanding the mechanism by which gut pathogens mediates immune-related disorders might be the solution to treat AIDs.

In this review, the relationship of the gut microbiota with immune system is covered along with the role of gut microbiota in certain AIDs. The pathogenic microbiota can induce Th1 and Th17 polarization, which can activate pro-inflammatory, self-reactive T cells that leads to onset of AIDs; Moreover, in AIDs, there is reduction in healthy microbiota that can provide regulatory, anti-inflammatory metabolites which promotes Treg induction. Restoring a healthy microbiota using targeted therapies holds promising avenue to manage AIDs. For example, probiotic treatments with *Bifidobacterium* and *Lactobacillus* strains can reduce the inflammation and increase Treg cells production. In addition, diet plan modification to produce SCFAs, like providing fiber-rich diet or supplementation with SCFA-producing bacteria has shown to decrease the disease severity. Fecal microbiota transplantation (FMT) has shown efficacy in mitigating the AIDs like IBD. Establishing causal relationships between specific microbial strains, their metabolites and AIDs through longitudinal studies like multi-omics approaches, including metagenomics, metabolomics, and transcriptomics should be the priority of future studies. Moreover, understanding every gene, metabolite, and protein in the microbiome that underlies intricate relationships with the immunological activities discussed here is still in its early stages. We have just begun to comprehend these relationships, and understanding every aspect of AIDs requires more comprehensive research. Above all, exploring this frontier in microbiota research not only promises to highlight the underpinnings of immune dysregulation but also to revolutionize the treatment paradigms for autoimmune diseases in future.
